# How anorexia nervosa patients with high and low autistic traits respond to group Cognitive Remediation Therapy

**DOI:** 10.1186/s12888-016-1044-x

**Published:** 2016-09-29

**Authors:** Kate Tchanturia, Emma Larsson, James Adamson

**Affiliations:** 1 King’s College London, Division of Psychological Medicine, Institute of Psychiatry, Psychology and Neuroscience, London, SE5 8AF UK; 2 Eating Disorders Unit, South London and Maudsley NHS Foundation Trust, Beckenham, UK; 3 Illia State University Tbilisi, Tbilisi, Georgia

**Keywords:** Anorexia nervosa, Autism spectrum disorders, Cognitive flexibility, Central coherence, Group therapy, CRT

## Abstract

**Background:**

The current study aimed to evaluate group Cognitive remediation therapy (CRT) inpatients with Anorexia Nervosa (AN). We aimed to examine the treatment response of group CRT in AN patients with high or low levels of autistic traits.

**Methods:**

Thirty-five in patients with an AN diagnosis received group CRT intervention for 6 sessions in a national eating disorder unit. All participants completed self-report questionnaires on thinking styles and motivation before and after the intervention.

**Results:**

Patients with low autistic traits had statistically significant medium size effect improvements in self-reported thinking style scales as well as confidence (ability) to change. Patients with high autistic traits showed no statistically significant improvements in any outcome measure.

**Conclusions:**

The brief group format CRT intervention improves self-reported cognitive and motivational aspects in people with AN without autistic traits. For patients with higher autistic traits brief group CRT does not improve self-reported cognitive style or motivation. This finding suggests that brief group format CRT might not be the best suited format for individuals with elevated autistic traits and individual or more tailored CRT should be explored.

## Background

Recent evidence on the cognitive features of anorexia nervosa (AN) suggests problems in cognitive flexibility (ability to shift between strategies and adapt to changes in the environment) and central coherence (attention bias to detail vs bigger picture) [for large database and systematic reviews see [9, 21, 22]], Poor cognitive flexibility and weak central coherence are the most commonly reported cognitive characteristics for autism spectrum disorders (ASD) [systematic review see [27]]. Similarities between AN and ASD was first reported in Gillberg and colleague’s studies (e.g. [6]) and further supported with evidence from the last three decades [4, 8, 11, 28] in neuropsychological, social cognitive and other domains.

Cognitive Remediation therapy (CRT) for AN was adapted to target cognitive flexibility, bigger picture thinking, perfectionistic thinking styles, encourage reflection on thinking styles and use awareness of cognitive styles in real life situations. CRT in individual and group formats was developed for adults and young people as well as family interventions using cognitive training principles (e.g. [10, 25]). The Individual CRT format recently gained more evidence from randomised treatment trials consistently showing cognitive improvements in patients receiving CRT as well as broader aspects of recovery [24, 25]. CRT in group format is less studied and no randomised treatment studies are available at present (for more details see Table 1).

**Table 1:** Published studies using the group format CRT in the field of eating disorders

Authors & publication date	Journal	Number of participants & Age group	Outcome measures	Main findings	Effect sizes
(Tchanturia, Larsson & Brown, 2016) [23]	Neuropsychiatry	42 completed DFlex, 77 CFS self-report questionnaires Age range: 17–59. 6 sessions.	Detail and Flexibility Questionnaire (DFlex) [16]. Cognitive Flexibility Scale (CFS) [12]	Significant improvement in the patients’ self-reported cognitive flexibility and bigger picture thinking, as well as in self-reported ability to change.	Dflex Rig *d* = 0.36, Dflex Det *d* = 0.37, CFS *d* = 0.18, MR Abi *d* = 0.34
(Lang et al., 2015) [10]	Psychiatry Research	6 female patients and 6 mothers, One off group to support the parents and patients, Mix adult/adolescents. AN Age range: 14–32.	Neuropsychological measures (Wisconsin card sorting task; The Rey Osterrieth Complex Figures Test) and Qualitative interview post-completion.	Overall positive feedback from the qualitative interview. Statistical analysis was not conducted, but an improvement on both patients and mothers neuropsychological scores post-intervention was observed.	N/A
(Asch et al., 2014) [2]	Encephale	10 adolescents (Final: 2). Age range: 12–17. Inpatients. 10 sessions.	Clinical inventories and neuropsychological assessments (WCST, TMT, Brixton)	Statistical analysis not conducted-improvement on most neuropsychological tests and clinical inventories.	N/A
(Zuchova, Erler & Papezova, 2013) [30]	Eating and Weight Disorders	2 groups 14 and 20 patients each. Adults. Inpatients. (33:1 F:M). Age range:16–35. 10 sessions .	No formal outcomes assessed. Observational study reporting positive evaluation from patients and facilitators.	Positive feedback from patients and facilitators. Patients able to reflect more post-treatment and tolerate own mistakes.	N/A
(Pretorius et al., 2012) [15]	European Eating Disorder Review	30 adolescents (29:1 F:M). (Final sample: 24). Day patients. Age range: 12–17. 4 sessions.	Cognitive flexibility Scale (CFS). Motivational Ruler (MR)	No significant differences on the CFS or Motivational ruler.	CFS *d* = 0.1, MR Imp *d* = 0.1, MR Abil *d* = 0.05
(Wood, Al-Khairulla & Lask, 2011) [29]	Clinical Child Psychology/Psychiatry	9 adolescents. Age range 13–19. One off group. 10 sessions.	No formal outcomes assessed. Observational study reporting positive evaluation from patients and facilitators	Participants appeared more aware of individual cognitive styles. Visible improvements in performance on some tasks.	N/A
(Genders & Tchanturia, 2010) [5]	Eating and Weigh Disorders	30 adults. (28:2 F:M). Age range: 14–60. Pilot group. 4 Sessions.	CFS, Rosenberg Self-Esteem Scale (RSE), MR.	MR Ability to change significantly improved. No significant change in CFS or RSE. Positive feedback from patients.	MR Ab *d* = 0.5, MR Imp *d* = 0.06, CFS *d* = 0.05, RSE *d* = 0.1

*Dflex* detail and flexibility questionnaire, Cognitive Rigidity and Attention to Details subscales; *CFS* cognitive flexibility scale, *MR* motivational ruler, Importance to change and Ability to change subscales; d-effect size

From the published literature, variety of the session length and content as well as outcome measures makes it difficult to draw systematic conclusions [23] and further research is needed.

In this study we aimed to examine the effects of CRT in an adult inpatient programme for AN patients with and without ASD traits.

## Methods

### Participants

Thirty-five participants with a diagnosis of AN based on DSM-5 from one of the consultant psychiatrists at the national eating disorder adult service were included in our study. Participants took part in a CRT group called “Flexibility workshop” (this title was selected by inpatients on the ward and we have kept this title to respect patients choice). Cognitive flexibility is one of the main target areas for CRT and the flexibility workshop represents an important part of the work happening in the group. The group runs on an alternating basis with other psychological groups offered on the ward, therefore participants are at various stages of their treatment when offered to attend the group.

### The CRT group (“Flexibility workshop”)

Data has been collected from multiple runs of the CRT group at an inpatient adult service for patients with severe AN. The groups are delivered by a multidisciplinary team with two facilitators per group, one of the facilitators is always a psychologist trained and supervised by the first author.

All sessions include practical, experiential exercises followed by reflection and discussion as well as psychoeducation about how the brain works, what we know about cognitive styles in AN and why we think flexibility workshop is useful in the recovery journey for patients [20]. Patients are also asked to plan their homework in the session together with facilitators. The facilitators aim to take a motivational and collaborative stance, by taking part in all the group exercises and planning of the homework tasks. The group explore different thinking styles, highlighting that there are pros and cons for each way of thinking but no right or wrongs. The first session involves psychological education about the brain and what research tells us about cognitive styles in eating disorders. The following sessions focus on multitasking, cognitive flexibility and bigger picture thinking, whereas the last session is focused on summarising the group by creating mind maps and relating the group content to the bigger picture of recovery. The group sessions in detail are described in the book outlining present research evidence and empirical findings [19, 23] and the clinicians manual [http://www.katetchanturia.com/publications].

### Procedure

All patients admitted to South London and Maudsley NHS Foundation Trust’s inpatient service were welcome to take part in the group. All participants were given information about the group and signed consent forms. All procedures performed in studies involving human participants were in accordance with the ethical standards of the institutional research committee and with the 1964 Helsinki declaration and its later amendments or comparable ethical standards.

The group consisted of 6 weekly sessions, and each session lasted for an hour. Patients were asked to complete the questionnaires before starting the group and again after the final session with the satisfaction questionnaire completed at the end of the group. Each group was attended by a mean of 5 participants.

Self-report Measures were chosen to measure targeted cognitive aspects in the group intervention and included:

#### Detail and flexibility questionnaire (DFlex) [16]

The scale consists of 24 items assessing cognitive rigidity and attention to detail. The clinical cut off for the cognitive rigidity subscale is 53 and above, and 44 for the attention to detail subscale. The scale has displayed high internal reliability and construct validity in both subscales [16].

#### Motivational ruler (MR) [13]

The questionnaire consists of 2 items measuring participants’ self-rated importance and ability to change. The scores are ranging from 0 to 10 with higher scores indicate greater importance or ability respectively. The measure has been used successfully in previous evaluation of individual and group work.

#### Patient feedback questionnaire

After completing the group, patients were also given a satisfaction questionnaire where they were asked to rate on a 5-point Likert scale whether they found sessions enjoyable, useful, had learnt any new skills and their opinion about the length of the group. In addition to the 4 quantitative questions, there were 3 qualitative questions asking the patients what they liked most about the sessions, what could be improved, and other groups they had attended.

#### ASD measures

Participants were assessed by at least one of the following measures; Autism-Spectrum Quotient (AQ): AQ-10 [1] a 10 item self-report questionnaire or the Autism Diagnostic Observation Schedule (ADOS). ADOS is a semi-structured clinical assessment tool which was conducted by a trained researcher as part of a separate study conducted on the ward, whereas the AQ questionnaire is a self-reported measure routinely administered as part of patients’ admission package. The patients’ scores have been separated in to low and high groups according to the clinical cut-off scores for each measure 23 % of participants (*N* = 8) were not assessed with clinical interview ADOS and in this cases only self-report AQ was used.

### Data analysis

Forty-nine patients took part in the first session of the group. 3 (6 %) of patients dropped out from the group; 11 patients (22 %) failed to complete or return both sets of questionnaires (these patients attended a different number of sessions). The remaining data (72 %) was examined for normality and afterwards each of the outcome measures from the first and final sessions of the group were analysed with paired t-tests using SPSS version 23. Repeated measures t-tests were then used to compare the between group’s performance over time in each of the clinical outcome variables. Cohen’s d effect sizes were computed for the all outcome measures [3].

## Results

The mean age of the patients was 26.2 (*SD* = 7.7). Patient’s mean BMI at the first session was 15.8 (*SD* = 1.8). Mean age of onset 18.2 (*SD* = 6.8) with a mean illness duration of 8.6 years (*SD* = 6.5). There were no significant differences between the two groups on any of the demographic variables assessed.

Table 2 displays differences on the clinical outcome measures between patients scoring high or low on the ASD measures.

**Table 2:** Evaluating differences in outcome measures between low and high scores on ASD measures

		First session		Last session			
Measures	n	Mean	SD	Mean	SD	p	d
*Low Scoring ASD*							
DFlex Cog Rig	21	54.5	9.7	50.5	8.5	.007	0.5
DFlex Attn Det	21	51.0	9.8	47.2	9.0	.053	0.4
MR Importance	21	7.8	1.9	7.9	2.0	.450	0.1
MR Ability	21	5.3	2.5	6.4	1.9	.004	0.5
*High scoring ASD*							
DFlex Cog Rig	14	56.9	10.3	57.2	8.8	.905	0.0
DFlex Attn Det	14	54.5	11.5	53.4	7.7	.702	0.1
MR Importance	14	7.1	3.0	7.6	3.0	.427	0.2
MR Ability	14	3.0	2.7	3.6	3.5	.389	0.2

*n* number of participants, *Dflex* detail and flexibility questionnaire, Cognitive Rigidity and Attention to Details subscales, *CFS* cognitive flexibility scale, *MR* motivational ruler, Importance to change and Ability to change subscales

**Fig. 1:**
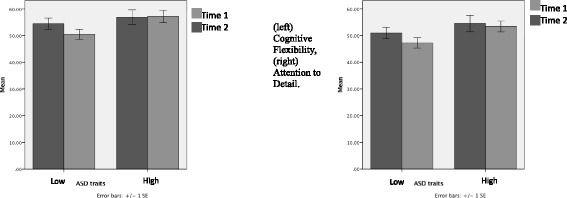
Repeated measures T-tests: cognitive flexibility and attention to details subscales

Paired t-tests revealed that there were significant differences on the Cognitive Rigidity subscale of the DFlex in the low scoring ASD group, with a medium effect size after CRT group (*p* > 0.01, *d* = 0.5) and trend on attention to detail scale with small effect size. Furthermore, there was also a significant medium effect size difference observed on patients self-reported ability to change, in the low scoring ASD group (*p* > 0.01, *d* = 0.5). However, there were no significant differences on any of the scales within the high scoring ASD group with negligible effect sizes after brief group CRT. There were no significant differences in patients’ evaluation of the group between high and low scorers. In addition, there were no significant differences on the magnitude of change between the two groups on the Dflex cognitive flexibility or attention to details subscales [F (1,33) = 2.19, *p* > .05; F (1,33) = 3.32, *p* > .05, respectively], or participants’ motivation to change [F (1,32) = 1.47, *P* > .05]. However, a significant difference on the magnitude of change between the two groups was observed for participants’ self-reported ability to change [F (1,32) = 6.59, *p* = .02]. Figure 1 illustrates patients scores over time between the low and high ASD trait groups self-reported cognitive flexibility and attention to detail subscales.

## Discussion

Our main question in this small scale naturalistic case series was to explore similarities and differences in treatment response in patients with anorexia nervosa with and without ASD traits receiving CRT group therapy as part of their inpatient admission. Patients’ self-reported cognitive style, motivation to change and their feedback on the intervention were used as outcome measures.

From the previous literature, as presented in Table 1, group CRT was reported in different clinical settings (mostly case series and clinical observations) but no research to date has been conducted to explore the impact of group CRT in patients with both AN and ASD. This is an important question because research evidence shows clear links between AN and ASD (e.g. [4, 6, 8, 14, 26, 27]). Amongst other similarities between AN and ASD, set shifting and bigger picture thinking are well researched and since CRT targets cognitive style the group setting is an ideal and safe environment to practice experiential cognitive exercises and role plays. It is therefore a very relevant question to explore similarities and differences in response to CRT group therapy for these patient groups. In addition to this, inpatient treatment for severe and enduring AN needs more development in the area of psychological treatment according to multicentre studies e.g. [7].

**Fig. 2:**
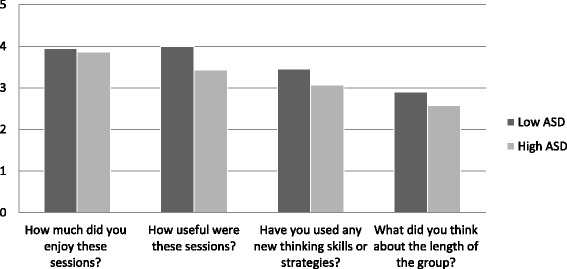
Patient feedback questionnaire

Our findings from this observational study does not allow us to draw causal conclusions about the effect of group CRT, due to a lack of control group, but instead provides interesting observations and pilot data for future studies. For example, our findings suggest that the subgroup of inpatients with AN who had no ASD traits reported medium size statistically significant improvements in self perceived flexibility of thinking and seemed to have developed a bigger picture approach after the group CRT. Their self-reported ability to change was also higher after the intervention, interestingly this increase was significantly larger than the small increase found in the high ASD trait group. The feedback questionnaire showed overall satisfaction with the brief group format from both groups, with no significant differences in feedback between the groups (Fig. 2).

This is an interesting finding as gaining insight into your own thinking patterns and being able to make changes and adaptations from a very early stage of treatment in an inpatient programme is helpful to engage patients in the treatment and make behavioural changes in different domains. In the context of inpatient psychological treatment, group interventions are practical and easy to deliver alongside nutritional and other inpatient treatment interventions [for more details see [18, 20] ‘Brief group psychotherapy for eating disorders: inpatient protocols’].

Our results also highlight that in the group of patients scoring high on one of the ASD measures (*N* = 14), no significant changes in self-reported thinking styles after group CRT or improvements in motivation; negligible effect sizes suggest that it is unlikely due to the power of study. From these results we can see that the current brief group format of CRT had no influence on self perceived cognitive styles. It is possible that the group setting, dosage or even style of delivery of the cognitive training could be modified for people with both AN and ASD traits.

In regards to the form of delivery of CRT, in AN literature the authors always highlight the importance of a motivational approach using Socratic questioning throughout the sessions moving from experiential exercises to reflective and implementation stages. This approach might be modified and adapted to suit an ASD/AN patient group making questions more concrete and giving more examples rather than expecting patients to do too much guess work and struggle with open ended questions. A recent systematic review of the CBT literature in ASD [17] has highlighted the need of protocol modifications for individuals with ASD. The authors presented useful recommendations to make CBT more accessible for the ASD group, similar adaptations are achievable for CRT.

Although this small case study is limited, it is informative and has useful implications when considering future developments, for example individual format of cognitive training will perhaps be more suited for people with comorbid ASD traits and AN. In future CRT studies, it is also worth exploring the proportion of comorbid ASD in AN samples, as the literature has shown that it is harder to diagnose women with ASD. Careful examination and clinical interviews will help detect a larger proportion of women in clinical settings with comorbid AN and ASD e.g. [11]. According to our results, inclusion or exclusion of an AN/ASD group might change results of other studies in the context of treatment evaluation. Our results suggest that the sub-group of patients with elevated ASD traits may need special adaptations of the available treatments in terms of length and content, not only to restore their nutrition, but to address wider aspects of recovery including addressing underlying anxiety and broader social functioning.

Our small observational study has a number of limitations for example the study was based on audit and a control group (non CRT group) will be needed in the future. Neuropsychological testing would also help to address the question around whether changes in thinking styles in the current study are subjective or reflective of improved cognitive test performance. It would also be worth addressing the role of co-morbidities, such as depression and anxiety, on cognitive change and treatment outcomes.

## Conclusions

This small study has implications for future studies for example, it will be easier to design studies with a more accurate power calculation, taking into account ASD traits by developing a modified version of group CRT for the individuals with AN and ASD.

## Abbreviations

ADOS: Autism diagnostic observation schedule
AN: Anorexia nervosa
AQ: Autism-spectrum quotient
ASD: Autism spectrum disorder
CRT: Cognitive remediation therapy
DFlex: Detail and flexibility questionnaire
MR: Motivational ruler

## References

[1] Allison C, Auyeung B, Baron-Cohen S (2012). Toward brief “Red Flags” for autism screening: The Short Autism Spectrum Quotient and the Short Quantitative Checklist for Autism in toddlers in 1,000 cases and 3,000 controls [corrected]. J Am Acad Child Adolesc Psychiatry.

[2] Asch M, Esteves J, De Hautecloque D, Bargiacchi A, Le Heuzey MF, Mouren MC, Doyen C (2014). Évaluation d’ un programme de remédiation cognitive au sein d’ un groupe d’ enfants et ¸ ais : étude d’ adolescents anorexiques franc exploratoire Cognitive remediation therapy for children and adolescents. Encéphale.

[3] Cohen J (1988). Statistical Power Analysis for the Behavioral Sciences.

[4] Courty A, Maria AS, Lalanne C, Ringuenet D, Vindreau C, Chevallier C, Berthoz S (2013). Levels of autistic traits in anorexia nervosa: a comparative psychometric study. BMC Psychiatry.

[5] Genders R, Tchanturia K (2010). Cognitive remediation therapy (CRT) for anorexia in group format: a pilot study. Eat Weight Disord.

[6] Gillberg C (1983). Are autism and anorexia nervosa related? The British Journal of Psychiatry. J Ment Sci.

[7] Goddard E, Hibbs R, Raenker S, Salerno L, Arcelus J, Boughton N, Treasure J (2013). A multi-centre cohort study of short term outcomes of hospital treatment for anorexia nervosa in the UK. BMC Psychiatry.

[8] Huke V, Turk J, Saeidi S, Kent A, Morgan JF (2013). Autism spectrum disorders in eating disorder populations: a systematic review. Eur Eat Disord Rev.

[9] Lang K, Lopez C, Stahl D, Tchanturia K, Treasure J. Central coherence in eating disorders: An updated systematic review and meta-analysis. The World Journal of Biological Psychiatry: The Official Journal of the World Federation of Societies of Biological Psychiatry. 2014:1–13. http://doi.org/10.3109/15622975.2014.909606. 10.3109/15622975.2014.909606 24882144

[10] Lang K, Treasure J, Tchanturia K (2015). Acceptability and feasibility of self-help Cognitive Remediation Therapy for anorexia nervosa delivered in collaboration with carers: a qualitative preliminary evaluation study. Psychiatry Res.

[11] Mandy W, Tchanturia K. Do women with eating disorders who have social and flexibility difficulties really have autism? A case series. Molecular Autism. 2015;6:6.http://doi.org/10.1186/2040-2392-6-6 10.1186/2040-2392-6-6 PMC4459459 26056560

[12] Martin MM, Rubin RB (1995). A new measure of cognitive flexibility. Psychological Reports.

[13] Miller WR, Rollnick S (2002). Motivational Interviewing: Preparing People for Change.

[14] Oldershaw A, Treasure J, Hambrook D, Tchanturia K, Schmidt U (2011). Is anorexia nervosa a version of autism spectrum disorders?. Eur Eat Disord Rev.

[15] Pretorius N, Dimmer M, Power E, Eisler I, Simic M, Tchanturia K. Evaluation of a cognitive remediation therapy group for adolescents with anorexia nervosa: Pilot study. European Eating Disorders Review. 2012. http://doi.org/10.1002/erv.2176. 10.1002/erv.2176 22488792

[16] Roberts ME, Barthel FM-S, Lopez C, Tchanturia K, Treasure JL (2011). Development and validation of the Detail and Flexibility Questionnaire (DFlex) in eating disorders. Eat Behav.

[17] Spain D, Sin J, Chalder T, Murphy D, Happé F (2015). Cognitive behaviour therapy for adults with autism spectrum disorders and psychiatric co-morbidity: A review. Research in Autism Spectrum Disorders.

[18] Sparrow KA, Tchanturia K. Inpatient Brief Group Therapy for Anorexia Nervosa: Patient Experience. International Journal of Group Psychotherapy. 2016. http://doi.org/10.1080/00207284.2016.1156406 10.1080/00207284.2016.1156406 38449129

[19] Tchanturia K (2014). Cognitive Remediation Therapy (CRT) for Eating and Weight Disorders.

[20] Tchantura K, Baillie C, Tchaturia K (2015). Recovery/Discovery Orientated Group. Brief group psychotherapy for Eating Disorders: Inpatient protocols.

[21] Tchanturia K, Davies H, Roberts M, Harrison A, Nakazato M, Schmidt U, Morris R. Poor cognitive flexibility in eating disorders: Examining the evidence using the wisconsin card sorting task. PLoS ONE. 2012. http://doi.org/10.1371/journal.pone.0028331 10.1371/journal.pone.0028331 PMC3257222 22253689

[22] Tchanturia K, Harrison A, Davies H, Roberts M, Oldershaw A, Nakazato M, Treasure J. Cognitive flexibility and clinical severity in eating disorders. PLoS ONE. 2011;6(6). http://doi.org/10.1371/journal.pone.0020462 10.1371/journal.pone.0020462 PMC3115939 21698277

[23] Tchanturia K, Larsson E, Brown A (2016). Benefits of group cognitive remediation therapy in anorexia nervosa: case series. Neuropsychiatrie: Klinik, Diagnostik, Therapie Und Rehabilitation: Organ Der Gesellschaft Osterreichischer Nervenarzte Und Psychiater.

[24] Tchanturia K, Lloyd S, Lang K (2013). Cognitive remediation therapy for anorexia nervosa: Current evidence and future research directions. Int J Eat Disord.

[25] Tchanturia K, Lounes N, Holttum S (2014). Cognitive remediation in anorexia nervosa and related conditions: a systematic review. Eur Eat Disord Rev.

[26] Tchanturia K, Smith E, Weineck F, Fidanboylu E, Kern N, Treasure J, Baron Cohen S (2013). Exploring autistic traits in anorexia: a clinical study. Molecular Autism.

[27] Westwood H, Eisler I, Mandy W, Leppanen J, Treasure J, Tchanturia K (2016). Using the Autism-Spectrum Quotient to Measure Autistic Traits in Anorexia Nervosa: A Systematic Review and Meta-Analysis. J Autism Dev Disord.

[28] Westwood H, Stahl D, Mandy W, Tchanturia K (2016). The set-shifting profiles of anorexia nervosa and autism spectrum disorder using the Wisconsin Card Sorting Test: a systematic review and meta-analysis. Psychol Med.

[29] Wood L, Al-Khairulla H, Lask B (2011). Group cognitive remediation therapy for adolescents with anorexia nervosa. Clin Child Psychol Psychiatry.

[30] Zuchova S, Erler T, Papezova H (2013). Group cognitive remediation therapy for adult anorexia nervosa inpatients: First experiences. Eat Weight Disord.

